# Sustainable Emerging Proteins: Allergenic Proteins in Edible Insects, Microalgae, and Microorganisms, and Desensitization Processing Technologies

**DOI:** 10.3390/foods15010069

**Published:** 2025-12-25

**Authors:** Fei Xu, Yan Zhao, Zhaowei Han, Xiaoyue Zhang, Bingyu Chen, Xuchun Zhu, Hongzhi Liu

**Affiliations:** 1 Heilongjiang Feihe Dairy Co., Ltd., C-16, 10A Jiuxianqiao Rd., Chaoyang, Beijing 100015, China; 2 School of Food and Health, Beijing Technology and Business University, Beijing 100080, China; 3 School of Food and Pharmaceutical Engineering, Guizhou Institute of Technology, Guiyang 550003, China

**Keywords:** allergens, edible insects, microbial protein, processing, allergy risk

## Abstract

As the global population continues to expand and demand for protein increases, alternative proteins (e.g., edible insect proteins, microalgae proteins, fungal or bacterial proteins) have emerged as a significant area of research interest due to their high nutritional value and sustainability. However, these novel protein sources may contain allergenic components, such as tropomyosin and arginine kinase in insects, phycocyanin in microalgae, and ribosomal proteins in fungi, which may trigger allergic reactions and cross-reactivity with traditional allergens. In this review, we systematically retrieved published studies from databases including PubMed and Web of Science, employing keywords such as microbial proteins, edible insects, and allergenicity. Articles were screened based on their relevance to allergenic properties and processing effects, with selected studies subjected to thematic analysis. The present paper reviews the allergenic properties of edible Insects, microalgae, and microorganisms’ proteins and their molecular mechanisms, and explores the effects of various processing techniques (e.g., heat treatment, enzymatic hydrolysis, high-pressure treatment, and glycosylation) on the reduction of allergenic activity. It was determined that the impact of processing methodologies is contingent on protein structure, with certain techniques having the potential to augment sensitization through epitope exposure. Furthermore, there are still gaps in the current research on the reduction in allergenicity of microbial and algal allergens, and future research should focus on the in-depth characterization of allergenic protein structures and the development of novel sensitization reduction techniques. This review provides a significant reference point for the safe development and rational application of edible insects, microalgae, and microorganisms proteins, which is of great importance for the development of sustainable food systems.

## 1. Introduction

Protein is the main bearer of life activities in the human body and is essential for growth, development, repair and maintenance of immune function. As the world’s population increases year by year, the demand for protein is growing. According to reports, the global population is projected to increase to 9.1 billion by 2050, with per capita daily calorie intake expected to rise from 2772 kcal to 3070 kcal [1].Currently, proteins can be divided into five categories according to their origin: proteins of animal origin (milk, eggs, fish, meat and animal offal), proteins of plant origin (pulses, cereals, oilseeds, etc.), edible insects, micro-algae and unicellular proteins (fungi, yeasts and bacteria). Of these, plant-derived proteins play an important role in the protein supply, with animal-derived proteins being the most expensive due to, inter alia, long economic cycles [2,3].Traditional protein production, which relies mainly on cultivation and farming, is highly land- and water-dependent, making it difficult to meet the growing demand for protein. Given the current shortage of plant and animal proteins, the development of alternative sources of protein has become a priority [4].

To date, a variety of alternative protein sources have been developed, such as: edible insect proteins, microalgae proteins, and single cell proteins (SCPs). Edible insects, with a protein content comparable to that of animal-origin protein sources and with all the essential amino acids, are now being used as an ideal alternative or supplement to traditional protein sources [5]. Unicellular organisms and microalgae are not only comparable to traditional protein sources in terms of amino acid composition and protein content, but also require significantly less water and cultivated area [6,7]. Thus, single-cell proteins and microalgae proteins are also important components of novel alternative proteins. Although, consuming enough protein is very important for the human body, consuming proteins along with allergenic proteins that can cause allergies can be fatal [8].

**Figure 1:**
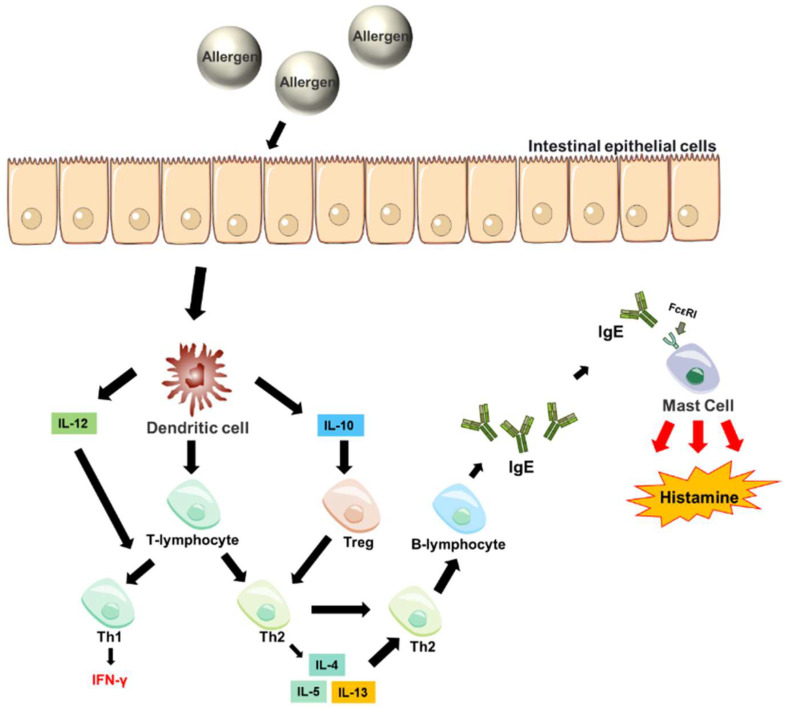
Causes of food allergy. After an allergen enters the body, it is recognized by effector T-cells and differentiates into immune B-cells that can release IgE. The IgE secreted by the B-cells binds to receptors on mast cells and is activated on a second exposure to the antigen. The activated mast cells release substances such as histamine leading to the development of an allergic reaction [11].

Food allergy is a condition that occurs when the body’s immune system mistakes a normally harmless food component (usually a protein) for a harmful substance, triggering a series of abnormal immune responses (Figure 1). It is estimated that food allergies affect approximately 5% of adults and 8% of children worldwide [9]. Symptoms of food allergy may involve the respiratory tract, gastrointestinal tract, skin and cardiovascular system [8]. Currently, the World Health Organization (WHO) and members of the International Union of Immunological Societies (IUIS) Allergen Nomenclature Sub-Committee have identified about 390 foods as allergenic (https://www.allergen.org). Eggs, milk, peanuts, nuts, wheat, and fish and shellfish are the most common foods that cause allergic reactions [10]. Although alternative proteins are not as popular in the daily diet nowadays compared to the food proteins mentioned above, allergic reactions caused by allergens in alternative proteins should be taken seriously.

Food allergies can somewhat impair the body’s absorption and utilization of a particular type of protein, which can only be supplemented by other protein sources. Fortunately, some forms of protein processing or modification can make allergenic proteins less allergenic, such as heat treatment, high pressure, glycosylation and fermentation [12,13,14]. In this review, we will focus on the allergenic proteins in these edible insects, microalgae, and microorganisms’ proteins and methods to reduce the allergenicity of the allergenic proteins, to provide guidance for consumers in selecting as well as protein products to reduce or avoid the occurrence of allergic reactions.

### 1.1. Edible Insect Proteins

**Figure 2:**
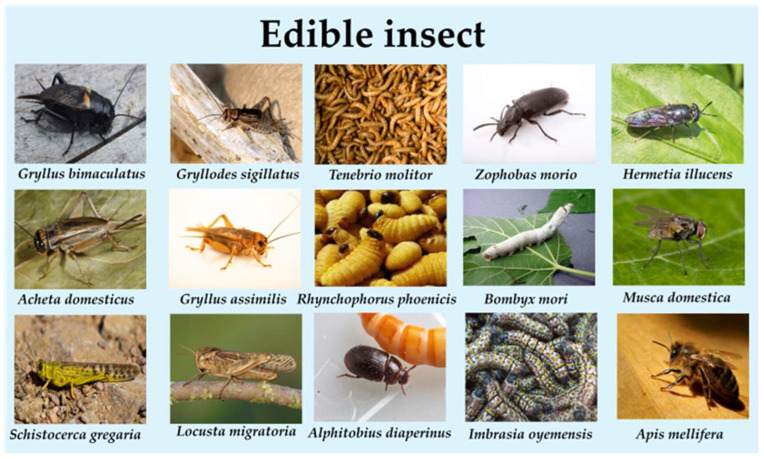
Fifteen Common Edible Insects and Their Scientific Names. The species represented include Coleoptera (e.g., *Rhynchophorus ferrugineus*), Lepidoptera (e.g., *Bombyx mori*), Orthoptera (e.g., *Gryllus bimaculatus*), and Hymenoptera (e.g., *Oecophylla smaragdina*).

Edible insects may refer to whole, dried and minimally processed insects, but may also refer to heavily processed powders, bars, artificial meat and fortified foods [15]. Edible insects are nutrient-dense, providing high-quality protein (up to 60% dry weight), essential amino acids, fats, and micronutrients (e.g., iron, zinc, B-vitamins) [16,17]. Insects consumed as food belong to diverse taxonomic orders (Figure 2), including Orthoptera (crickets, grasshoppers), Coleoptera (mealworms), and Lepidoptera (silkworms) [18]. Edible insects have a low dependence on land and water compared to traditional livestock, and a richer amino acid profile and higher protein content than plant protein sources, as well as simultaneous nutrients such as vitamins and minerals [4,19].

Beyond their nutritional profile, insect proteins also exhibit functional properties that enhance their potential as sustainable food ingredients. For example, edible insect protein has good water-binding, oil-binding, foaming and emulsifying properties, and is able to form functional peptides with health benefits (such as antioxidants, etc.) [20,21,22].

While insect protein is physiologically more acceptable than whole insects to some consumers, safety concerns remain. Heavy metals [23] (e.g., from contaminated environments) and natural toxins [24] (e.g., cyanide in certain species) may pose risks, as can pesticide residues if insects are harvested from treated areas. However, these hazards can be minimized through controlled breeding, feed regulation, and post-harvest processing.

### 1.2. Single-Cell Proteins

Cellular agriculture is an emerging field of biotechnology dedicated to the development of a variety of alternatives for commercial agricultural production, including food and feed that utilize microorganisms and microalgae as a source of high protein, collectively referred to as SCP. The development of microbial proteins is both a way to fill possible protein resource shortages and a green and sustainable way to grow. We can produce microbial proteins from by-products of the fermentation industry or agro-industry, e.g., waste brewer’s yeast recycled from the fermentation industry and photosynthetic bacteria (*Rhodopseudomonas* sp.) from biogas treatment processes [25,26]. However, issues such as high nucleic acid content and cost constraints have prevented large-scale production of microbial proteins for applications [27,28,29].

#### 1.2.1. Microalgae Proteins

Microalgae have been shown to contain a protein content that exceeds 50%, which is indicative of their high nutritional value. This has led to microalgae being identified as a promising source of non-animal proteins [30]. Phycocyanin from microalgae is a natural food colorant. Natural food coloring not only provides vibrant colors, but also has potential health benefits, such as: antioxidant, antibacterial, anticancer, hypolipidemic, etc. [31,32]. *Spirulina* is a significant source of phycocyanin for human consumption, attributable to its high phycocyanin content [33]. In addition to its high production of phycocyanin, *Spirulina* has become an important source of dietary supplements due to its nutrient-rich nature and is widely added to snacks, pasta, cookies, and breads, accounting for 99.5% of the total global production of microalgae [34]. *Spirulina* is rich in protein, up to 60–70% of dry weight, which is much higher than the protein content of meat (15–25%) and beans (35%) [35]. In addition to its high protein content, *Spirulina* is also high in macronutrients and micronutrients [36]. According to United Nations Educational, Scientific and Cultural Organization (UNESCO), “*Spirulina* is the perfect food for tomorrow” and the US Food and Drug Administration (USFDA) has called it “one of the best sources of protein” [36]. Despite the evident advantages of microalgae proteins in terms of sustainability and high nutritional value, there are still some disadvantages and shortcomings associated with their development as an alternative protein source. These include higher production costs, organoleptic properties and flavour, innovation in extraction and refining processes, and economic feasibility issues [37,38,39].

#### 1.2.2. Fungal, Bacterial and Yeast Proteins

Fungal or bacterial proteins are another great source of single-cell proteins. The feasibility, tolerance and metabolic effects of consuming fungal proteins were experimentally determined as early as the 1970s [40]. In addition to nutrients such as lipids and cell wall glucans, unicellular organisms have high protein content. In *Saccharomyces cerevisiae*, for instance, the protein content of yeast extracts can reach 50–70% of the dry weight [41]. Yeast proteins are highly bioavailable to humans and animals, with studies showing that yeast proteins are 96% digestible and 59% net utilizable [42]. Yeast cell proteins have a higher protein efficiency ratio (PER) compared to proteins from conventional sources. In addition, yeast proteins are a rich source of bioactive peptides, which can exert positive effects on human health [41,43]. Another widely used fungus is *Fusarium venenatum*. *F. venenatum* has a protein content of 45–54% dry weight and is now often used to produce meat substitutes because of its meat-like structure [42,44,45]. The proteins of *F. venenatum* are highly similar to yeast proteins in composition and characterization, with digestibility-corrected amino acid scores comparable to milk proteins and higher than those of chicken versus beef, making it a very promising alternative protein source [46]. Bacterial proteins are also a commonly used single-cell proteins and are mostly used in animal feed. Bacterial proteins make up 50–80% of the dry weight and bacteria have shorter growth times and faster growth rates compared to fungi and yeast [45]. Moreover, it has been demonstrated that bacteria have the capacity to proliferate on a wide range of substrates, including gaseous substrates [47]. *Photosynthetic Bacteria* (PSB) are ancient microorganisms that are widely distributed in a variety of environments [48,49,50]. PSB is rich in microbial proteins and contains high value carotenoids, bacterial chlorophyll and CoQ10 [51,52,53]. Some studies have shown that photosynthetic bacteria can treat a variety of wastewater under the right conditions [54]. It has been demonstrated that PSBs are capable of producing proteins in a highly efficient manner from these wastewaters. Nevertheless, there are numerous challenges associated with the cultivation of PSB using wastewater. The primary issue that must be addressed pertains to the potential presence of pollutants in the wastewater, including heavy metal ions in industrial wastewater, disease-causing microorganisms in municipal wastewater, and pesticide residues in agricultural wastewater. This has a considerable impact on the production of PSB. Moreover, the question of consumer acceptability of wastewater farming represents a significant challenge that impacts the competitiveness of the PSB market. The large-scale development of microbial protein production has the potential to address two significant challenges: the scarcity of protein resources and the environmental impact of intensive livestock farming.

Despite the numerous advantages exhibited by single-cell proteins, which are analogous to microalgae proteins, their elevated nucleic acid content constitutes a pivotal concern. This high nucleic acid content exerts a substantial influence on the organoleptic properties and flavor of the proteins. Consequently, further processing is imperative prior to the extraction of the proteins. SCPs possess considerable potential as a sustainable protein source, exhibiting both sustainability and environmental friendliness. Nevertheless, further process and technology innovations are required in order to address issues such as sensory properties and cost. Should these challenges be surmounted in the future, SCP will indubitably play an important role in the food industry.

### 1.3. Applications of Insect Protein and Microbial Protein in Foods

As emerging protein sources, insect protein and microbial protein are gradually gaining attention within the food industry. Table 1 summarizes the relevant applications of insect protein and microbial protein in food products. Insect protein is rich in high-quality amino acids and offers advantages such as rapid reproduction and high feed conversion efficiency. Microbial protein, on the other hand, exhibits benefits including short growth cycles and the ability to be produced using waste materials. Furthermore, microbial protein can be processed through cell autolysis or enzymatic hydrolysis to obtain functional peptides (e.g., ACE inhibitory peptides, immunomodulatory peptides), thereby providing potential for the development of high-value food ingredients. These unique characteristics endow them with significant potential to meet future global protein demands, and their application in food systems has garnered increasing interest. In addition, the incorporation of edible insects or microbial protein into food products can enhance both the nutritional profile and physicochemical properties of the final food items.

## 2. Allergens

With the widespread use of alternative proteins, especially the continued development of new alternative protein sources such as insect proteins, microalgae proteins, and fungal or bacterial proteins, allergic reactions to these alternative proteins have attracted increasing attention from consumers. Despite the significant environmental and health benefits of these alternative proteins, they also exhibit unique characteristics in terms of the types of allergens they contain. Understanding the types of these allergens and the immune responses they elicit is critical to safeguarding consumer food safety and health. Allergenic proteins in common edible insects, microalgae, and microorganism proteins are shown in Table 2.

### 2.1. Edible Insect Allergens

In addition to the toxicity and heavy metal exceedances previously mentioned, which render insects less acceptable to consumers, allergic reactions caused by edible insect protein allergens represent a significant issue with regard to the consumption of insects [77]. Tropomyosin, arginine kinase have been identified as major allergens in insects [78]. In economically underdeveloped regions or countries, such as Africa, exposure to or consumption of insects is common, as insects provide the body with the protein and energy it needs on a daily basis. However, studies and reported cases of insect allergy in Africa are uncommon. This may be due to the fact that most studies have focused more on the nutritional value of insects to address problems such as malnutrition in Africa [79]. Whereas in China, there are frequently reported cases of allergy to edible insects [80,81].

#### 2.1.1. *Bombyx mori*

*Bombyx mori* and its derivatives are known for their high nutritional, medicinal and economic values [82]. The total protein, lipid content and ash (dry weight) of silkworm pupae were 71.9%, 20.1% and 4.0%, respectively [83]. In China, silkworm pupae are the most commonly consumed insect. However, it is estimated that more than a thousand people become allergic to silkworm pupae each year. Symptoms of silkworm pupa allergy include vomiting, dizziness and asthma [83].

Liu et al. cloned and expressed arginine kinase (Bomb m 1) from silkworm larvae and confirmed its allergenicity by methods such as ELISA and Western blotting [84]. It was found that silkworm arginine kinase binds to serum IgE from silkworm-allergic patients and that it is cross-reactive with arginine kinase from cockroaches. Tropomyosin is a recognised invertebrate pan-allergen and is no exception in the *Bombyx mori*. Tropomyosin (Bomb m 3) is an important allergen in silkworm pupae. By comparison with other known allergens, Tropomyosin from silkworm pupae showed 73.5% to 92.3% amino acid sequence homology, with particularly high similarity to Tropomyosin from cockroaches and dust mites [85]. *Bombyx mori* lipoprotein 3 (Bmlp3) is a 27.6 kDa allergenic protein defined as Bomb m 6 that elicits a strong IgE response in the sera of domestic silkworm allergy sufferers [86]. Bmlp3 is a major allergen in the domestic silkworm and is highly stable to heat, acid and enzyme digestion, meaning that it can still trigger allergic reactions in a wide range of food processing conditions. In addition, another major allergen in the silkworm is Hemolymph lipoprotein (Bomb m 4), which Jeong et al. identified by proteomic analysis and confirmed to be allergenic by ELISA [87]. Bomb m 9 is a structural protein present in the haemolymph of the silkworm, *Bombyx mori*, belonging to the 30k family, which binds to IgE in the serum of silkworm pupa-allergic individuals to cause allergic asthma [88]. However, unlike silkworm pupa arginine kinase, Bomb m 9 has no cross-reactivity with allergenic proteins in cockroaches. In addition to the allergenic proteins mentioned above, there are two other allergenic proteins, lipoprotein (Bomb m 5) and lipoprotein 3 (Bomb m 6), in the domestic silkworm (Table 2).

#### 2.1.2. *Tenebrio molitor*

*Tenebrio molitor* is an emerging edible insect. Freeze-dried *T. molitor* larvae contain about 33% fat, 51% crude protein and 43% true protein (on a dry matter basis) and are often added to pasta, energy bars and biscuits [89,90]. *T. molitor* protein extracts contain a variety of possible allergens including Actin, HSP70, α-amylase, Myosin light chain, Arginine kinase and Tropomyosin [78,91]. Among them, Tropomyosin and Arginine Kinase are known to be the main allergens of *T. molitor* [78]. Multiple allergens in *T. molitor* cross-react with other known arthropod (e.g., shrimp, crab, etc.) allergens [91]. The Allermatch website (https://allermatch.org/) allows comparative analysis of the sequences of target proteins and allergenic proteins. *T. molitor* Tropomyosin has more than 35% identity with known Tropomyosin that can cause allergies, such as shrimp Tropomyosin [91]. Therefore, people who are allergic to shrimp may also be allergic to *T. molitor* and should be vigilant when choosing edible insect foods [78,92].

#### 2.1.3. *Hermetia illucens*

*Hermetia illucens* is a potential sustainable and nutritious edible insect. The protein content of *H. illucens* can reach 40–60%, with a relatively low fat content of 10–30%, and they are rich in essential amino acids, fatty acids, and minerals such as calcium, iron, and zinc, making them an ideal raw material for healthy food [93]. However, at present, the utilization of *H. illucens* is mainly limited to animal feed, and it has not been approved by regulations as a food ingredient. This is because they mostly grow in garbage, and the decomposition of garbage and waste may cause *H. illucens* to accumulate a large amount of heavy metals and pollutants in their bodies. Eating *H. illucens* is bound to have an impact on human health. Nevertheless, it cannot be denied that if waste and other waste materials can be used reasonably to breed *H. illucens* as a food ingredient, it will be a green, sustainable and nutritious food source. The main allergens in *H. illucens* include Tropomyosin, Arginine Kinase, and Myosin light chain. Studies have shown that the Tropomyosin in *H. illucens* has a high degree of similarity (about 75–80% sequence similarity) with that in shrimp (such as *Penaeus monodon*), which means that people allergic to shrimp may also have allergic reactions to *H. illucens* [94]. In addition, through immunoblotting experiments, Tropomyosin is widely recognized in *H. illucens* extracts and shows strong IgE binding ability, especially for people allergic to seafood [94]. In addition, other allergenic proteins such as Troponin, Triosephosphate isomerase, Hemocyanin, Cuticle proteins, and Odorant-binding proteins have also been identified in *H. illucens*.

#### 2.1.4. *Periplaneta americana* & *Blattella germanica*

*Periplaneta americana* and *Blattella germanica* are two common cockroach species. *Periplaneta americana* is larger in size and prefers hot and humid environments, while *Blattella germanica* is smaller and more commonly found in dry and cool areas [95,96]. According to the WHO/IUIS website (https://www.allergen.org), both *P. americana* and *B. germanica* have been identified to possess over ten allergenic proteins (Table 2). Notably, there is a considerable degree of homology and cross-reactivity between the allergens of *B. germanica* and *P. americana* [97]. Studies have shown that Bla g 1 and Bla g 2 are the major allergens of *B. germanica*. These allergenic proteins bind to IgE in the serum of allergic patients, triggering allergic asthma, which is commonly observed in regions such as the United States [98,99]. Among them, Bla g 2 can also serve as a key biomarker for *B. germanica* exposure and allergy. In addition, Bla g 5, a Glutathione S-transferase (GST), is one of the most prevalent cockroach allergens in the US population [100]. Research has found that GST from different cockroach species exhibit low cross-reactivity, making this protein highly useful for accurate allergen diagnosis [97]. Tropomyosin in cockroaches (Bla g 7 and Per a 7) is a “pan-allergen” present in the muscle tissues of various organisms (mollusks, arthropods, parasites, etc.) and can induce IgE cross-reactivity [101]. Arginine kinase (Bla g 9 and Per a 9) is also a major cockroach allergen that binds strongly to IgE in patient serum [95]. As a cross-reactive allergen between shrimp and arthropods, Arginine Kinase in cockroaches also exhibits IgE cross-reactivity [102]. Studies have indicated a close association between cockroaches and allergic respiratory diseases, with a more severe situation observed among low-income urban populations [103]. In Taiwan, 58% of asthma patients are allergic to the Per a 2 allergen of cockroaches, and in Poland, approximately 25% of asthmatic children are sensitive to cockroaches [104,105]. Kangfuxin liquid (KFX) is a traditional Chinese medicine for promoting wound healing in China. It can be applied externally by soaking gauze in the medicinal liquid and applied to the affected area, or taken orally. The main component of KFX is an extract from *P. americana*. This may be one of the reasons why KFX is prohibited for use in individuals with asthma. For allergic asthma caused by cockroaches, immunotherapy for cockroach allergy is currently available, but the most effective approach remains avoiding exposure to cockroach-infested environments [106]. In addition to the aforementioned allergenic proteins, cockroaches also contain various other allergens such as hemolymph proteins, Troponin, Myosin Light Chain, serine proteases, α-Amylases, and chitinases (Table 2).

#### 2.1.5. Other Edible Insects

*Acheta domesticus* is another popular edible insect commonly used as a food ingredient in barbecues. The protein content of *A. domesticus* is 47%, with a fat content of 25%, and it is also rich in minerals and vitamins [107]. To date, various allergenic proteins have been identified in *A. domesticus*, including tropomyosin, Myosin heavy chain isoform, Myosin heavy chain, and Myosin Light Chain [108]. Tropomyosin is the major allergen in *A. domesticus*. Studies have shown that the actin in crickets shares significant amino acid sequence similarity with that in shrimp, particularly in the binding epitopes [109]. This similarity may lead to allergic reactions to crickets in individuals allergic to shrimp. A real-world prevalence study on IgE reactivity to crickets, locusts, and mealworms involving 2000 participants revealed that 9.7% (195 individuals) were sensitive to insects. Among these 195 individuals, 34% had common recognition of tropomyosin, and 18.5% tested positive for arginine kinase [110].

*Locusta migratoria*, belonging to the order Orthoptera and family Acrididae, comprise 6787 known species [111]. The protein content of locusts can reach 18–29%, comparable to or even higher than that of meat [112]. In addition to their high protein content, locusts are also rich in vitamins, minerals, amino acids, as well as high levels of sterols and unsaturated fatty acids [112]. Locusts have become a traditional food in some regions as part of the diet [113,114]. However, for certain populations, consuming locusts may trigger acute allergic reactions and even lead to anaphylactic shock [115]. Ji et al. reported 27 cases of anaphylactic shock caused by locust consumption over the twenty-year period from 1980 to 2008 [116]. The main allergenic proteins in locusts include arginine kinase and hemocyanin [114,117]. These two proteins are commonly found in various insects and crustaceans, thus potentially causing cross-reactions similar to seafood allergies. Wang et al. identified a hexamerin-2 protein in locusts that binds strongly to IgE in the serum of locust-allergic patients [118]. Nevertheless, when processing locusts, removing body parts such as wings, legs, and antennae and employing processing methods like grinding can reduce the risk of allergies caused by locusts [112].

#### 2.1.6. Prevention and Control of Allergy Risks from Edible Insects

**Figure 3:**
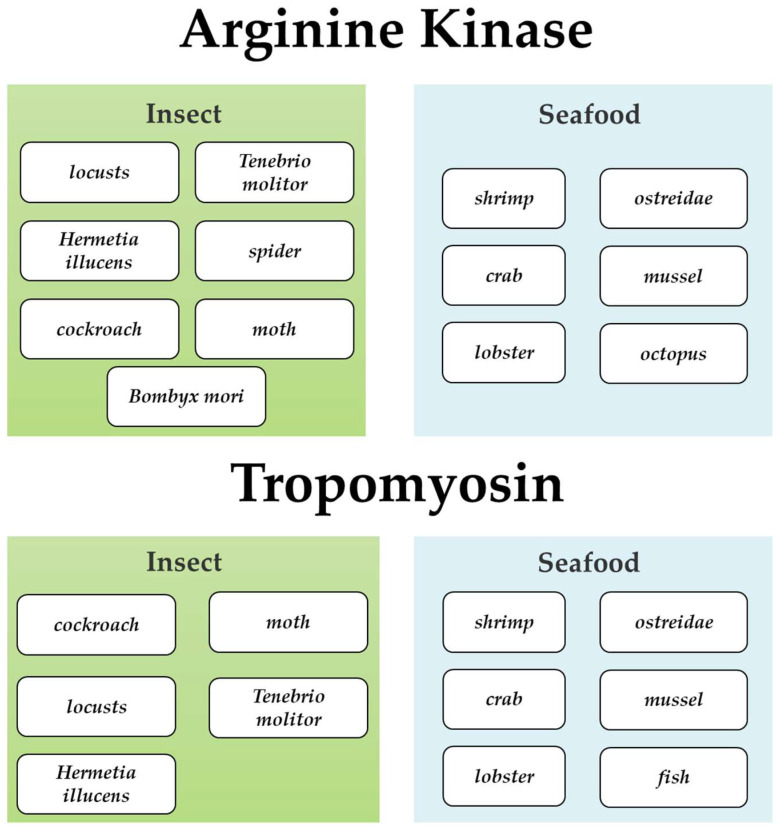
Distribution of allergenic proteins arginine kinase and tropomyosin in insect and seafood sources. The upper figure shows arginine kinase present in various insects and seafood. The lower figure displays tropomyosin in different insects and seafood products.

Edible insects, as an emerging sustainable protein source, possess high nutritional value and environmental friendliness; however, their potential allergenicity cannot be overlooked. Tropomyosin and Arginine Kinase are the most prevalent “pan-allergens” in edible insects, widely present in *B. mori*, *T. molitor*, *H. illucens*, *A. domesticus* and *L. migratoria* (Figure 3). These allergens not only have the potential to trigger local or systemic allergic reactions (such as rashes, vomiting, asthma, and even anaphylactic shock) but also exhibit significant cross-reactivity with allergens from crustaceans (e.g., shrimp, crab), mites, and cockroaches, thereby increasing the allergenic risk for individuals with seafood allergies or asthma. Therefore, when edible insects are used as food ingredients, potential allergenic risks should be clearly indicated on the packaging to inform consumers. For edible insect proteins, further development of low-allergenicity processing technologies is essential in the future to mitigate the risk of allergic reactions in sensitized individuals following consumption.

### 2.2. Microbial Allergens

The increasing integration of microorganisms into food production, pharmaceuticals, and environmental applications has revealed an underappreciated public health concern: microbial allergenicity. Unlike conventional allergens derived from pollen or animal dander, microbial allergens exhibit unique molecular features—including intrinsic thermostability, conserved functional domains, and frequent cross-reactivity across phylogenetically distant species—that complicate their detection and clinical management. These characteristics stem from the evolutionary conservation of proteins essential for microbial survival, which paradoxically render them both biotechnologically valuable and immunologically reactive. Despite the increasing evidence for the existence of microbial allergens, systematic studies on their molecular mechanisms and allergenic pathways are still limited, posing challenges to risk assessment and regulatory frameworks.

#### 2.2.1. Microalgae Allergens

As a highly diverse group of photosynthetic organisms, microalgae contain intracellular proteins such as phycocyanin that have been confirmed to induce allergic symptoms through IgE-mediated immune responses, clinically manifesting as respiratory inflammation, skin reactions, and even systemic anaphylaxis. Studies have demonstrated that phycocyanin is the primary allergen responsible for *Spirulina*-induced allergies [119,120]. As the main pigment protein in *Spirulina*, phycocyanin exhibits various biological effects, including antioxidant and anti-inflammatory properties. In addition to phycocyanin, six proteins in *Spirulina* extracts have been detected with sequence homology to allergenic proteins from other sources [34]. Computational and proteomic analyses have revealed that these proteins share sequence homology with food allergens such as corn, fish, and shrimp [121]. Therefore, individuals allergic to fish and shellfish should exercise caution when consuming *Spirulina*-enriched foods due to potential allergic risks.

Another microalga frequently reported to cause allergies is *Chlorella*, likely because it is more commonly used in health supplements alongside *Spirulina*. Allergenic proteins with molecular weights of 13, 17, 19, 25–26, 46–50, and 72 kDa have been identified in *Chlorella* [122,123]. Similarly, Mariachiara et al. conducted a computational assessment of sequence homology between *Chlorella* proteins and known allergens [120]. Four proteins were found to exhibit sequence identity with other allergenic proteins, among which fructose-bisphosphate aldolase was identified as the *Chlorella* protein most likely to cause cross-reactivity upon ingestion due to its significant sequence homology with the corresponding protein in edible fish and crustaceans. Consequently, individuals allergic to fish and crustaceans should be cautious about consuming health supplements containing algal additives to avoid potential allergic reactions.

#### 2.2.2. Fungal Allergens

The widespread application of microorganisms in environmental, food, and pharmaceutical fields has increasingly drawn attention to their potential allergenicity. Fungi, bacteria, and yeasts can produce various allergenic proteins capable of inducing specific IgE antibody production in the host immune system, thereby triggering allergic reactions. *Alternaria alternata*, a common outdoor fungus, is one of the major airborne allergens whose spores are significant contributors to allergic sensitization. It shows a strong association with allergic respiratory diseases, particularly asthma [124,125]. To date, numerous *A. alternata* allergenic proteins have been identified and purified (Table 2) [126]. Alt a 1 is the major allergen of *A. alternata*, with approximately 80% of patients allergic to this fungus showing sensitivity to this allergen [127,128]. Ribosomal protein P2 (Alt a 5) represents a minor allergen of *A. alternata* and shares sequence homology with the Ribosomal protein P2 found in *Fusarium culmorum* [129]. It is well established that cross-reactivity between proteins can arise from shared amino acid or carbohydrate epitopes [130]. Given the presence of Ribosomal protein P2 in both *A. alternata* and *F. culmorum*, cross-reactivity between these two species is highly plausible. In addition to Ribosomal protein P2 (Alt a 5), other allergens in *A. alternata* such as Heat shock protein 70 (Alt a 3), Enolase (Alt a 6), Glutathione-S-transferase (Alt a 13), and Manganese superoxide dismutase (Alt a 14) also exhibit potential for cross-reactivity with other known allergenic proteins.

*Saccharomyces cerevisiae*, an essential microorganism in food processing widely used in bread and beer production, may still induce allergic reactions despite inactivation during processing [131]. Among its allergenic components, enolase from *S. cerevisiae* has drawn particular attention as a major sensitizer due to its high secretory level and conserved structure. Baldo et al. evaluated the sensitization profile of 47 fungal inhalation allergy patients to *S. cerevisiae* through specific allergy testing [131]. The study revealed that 35 patients exhibited positive reactions to *S. cerevisiae* extracts, with immunoblot analysis confirming yeast enolase as the primary allergenic protein. RAST inhibition assays further demonstrated significant cross-reactivity between *S. cerevisiae* enolase and *Candida albicans* extracts. Clinically, a case of anaphylactic shock was reported in a fungal allergy patient following consumption of yeast-containing food products. Skin prick tests (SPT) and serum IgE measurements confirmed cross-reactive IgE antibodies against multiple fungi including *S. cerevisiae*. Additionally, allergen databases have identified several other potential allergens in *S. cerevisiae*, including glucosidase, β-fructofuranosidase, manganese superoxide dismutase (Mn-SOD), profilin, and ribosomal proteins (Table 2).

In addition to *S. cerevisiae*, another well-known commercial fungus is *Fusarium culmorum*, which is commonly utilized in the production of meat substitutes, specifically plant-based meats. *F. culmorum* contains three identified allergenic proteins: Ribosomal protein P2, Thioredoxin reductase, and transaldolase. Research has demonstrated that Ribosomal protein P2 (Fus c 1) present in the mycoprotein (Quorn) produced by *F. culmorum* serves as the primary allergen [132]. Fus c 1 exhibits high sequence homology with Ribosomal proteins from various molds, including Alt a 5 mentioned previously, which may lead to IgE cross-reactivity [129,132]. Furthermore, both *F. culmorum* and *S. cerevisiae* possess allergenic proteins of the same types found in algal proteins, suggesting potential sequence homology among these proteins. Consequently, individuals allergic to these fungal proteins may also be at risk of allergic reactions to algal proteins.

#### 2.2.3. Prevention and Control of Microbial Allergy Risks

The widespread application of fungi, bacteria, and yeasts in environmental, food, and pharmaceutical fields necessitates attention to their potential allergenicity. Microorganisms such as *A. alternata*, *S. cerevisiae*, and *F. culmorum* can produce various allergenic proteins that induce IgE-mediated allergic reactions and are closely associated with diseases such as asthma, allergic rhinitis, and even anaphylactic shock. Notably, homologous proteins across different microorganisms (e.g., Alt a 5 and Fus c 1, *S. cerevisiae* enolase and *C. albicans* protein extracts) may trigger cross-reactivity, thereby increasing the risk of allergic sensitization. This cross-sensitization is not only observed among fungi but may also extend to cross-reactivity with algal proteins.

**Table 2:** Allergenic proteins in edible insects and microalgae (“*” denotes formally registered allergenic proteins in the allergen nomenclature database, “**” denotes non-formally registered allergenic proteins in the allergen nomenclature database, Allergen Nomenclature Database Website: www.allergen.org).

Type	Source	Protein Type	Allergen	MW (kDa)	Reference
Edible insects	*Bombyx mori*	Arginine kinase	Bomb m 1 *	42	Yue et al. [86]
	*Bombyx mori*	Tropomyosin	Bomb m 3 *	40	Yue et al. [86]
	*Bombyx mori*	Hemolymph lipoprotein	Bomb m 4 *	30	Yue et al. [86]
	*Bombyx mori*	lipoprotein	Bomb m 5 *	30	Yue et al. [86]
	*Bombyx mori*	Apolipophorin-III	Bomb m 6 *	25–33	Yue et al. [86]
	*Bombyx mori*	30K Protein	Bomb m 9 **	30	Zuo et al. [88]
	*Acheta domesticus*	Tropomyosin	Unknown **	32–4	Lamberti et al. [108]
	*Acheta domesticus*	Myosin heavy chain isoforms	Unknown **	224.7	Kamemura et al. [133]
	*Acheta domesticus*	Myosin heavy chain	Unknown **	135	Lamberti et al. [108]
	*Acheta domesticus*	Myosin Light Chain	Unknown **	2	Lamberti et al. [108]
	*Acheta domesticus*	Troponin T	Unknown **	46.7	Lamberti et al. [108]
	*Acheta domesticus*	Hexamerin-like protein 2	Unknown **	75	de las Marinas et al. [134]
	*Pygmy grasshoppers*	Cuticle proteins	Unknown **	18.9	Lamberti et al. [108]
	*Pygmy grasshoppers*	Myosin heavy chain	Unknown **	222.8	Lamberti et al. [108]
	*Pygmy grasshoppers*	Myosin Light Chain	Unknown **	22.5	Lamberti et al. [108]
	*Pygmy grasshoppers*	β-Actin	Unknown **	41.7	Lamberti et al. [108]
	*Pygmy grasshoppers*	Troponin T	Unknown **	46.7	Lamberti et al. [108]
	*Pygmy grasshoppers*	Lysozyme	Unknown **	80.0	Lamberti et al. [108]
	*Pygmy grasshoppers*	Tropomyosin	Mec e 7 **	38	Leung et al. [135]
	*Locusta migratoria*	Hexamerins	Unknown **	78	Pharima et al. [136]
	*Locusta migratoria*	Arginine kinase	Unknown **	≈40	Egonyu et al. [112]
	*Locusta migratoria*	Hemocyanin	Unknown **	≈75	Egonyu et al. [112]
	*Locusta migratoria*	Cuticle proteins	Unknown **	12–17	Lamberti et al. [108]
	*Locusta migratoria*	Myosin Light Chain	Unknown **	23	Lamberti et al. [108]
	*Tenebrio molitor*	Tropomyosin	Ten m 7 **	35	Barre et al. [78]
	*Tenebrio molitor*	α-Amylases	Unknown **	≈50	Barre et al. [78]
	*Tenebrio molitor*	Arginine kinase	Unknown **	≈40	Barre et al. [78]
	*Tenebrio molitor*	Hexamerins	Unknown **	70	Barre et al. [78]
	*Tenebrio molitor*	α-tubulin	Unknown **	Unknown	Ribeiro et al. [91]
	*Tenebrio molitor*	β-tubulin	Unknown **	Unknown	Ribeiro et al. [91]
	*Tenebrio molitor*	Actin	Unknown **	41.9	van Broekhoven et al. [137]
	*Tenebrio molitor*	Fructose biphosphate aldolase	Unknown **	Unknown	Ribeiro et al. [91]
	*Tenebrio molitor*	Myosin Light Chain	Unknown **	21.7	Lamberti et al. [108]
	*Tenebrio molitor*	Troponin T	Unknown **	45.8	Lamberti et al. [108]
	*Hermetia illucens*	Hemocyanin	Unknown **	≈75	Karnaneedi et al. [109]
	*Hermetia illucens*	Tropomyosin	Unknown **	≈33	Karnaneedi et al. [109]
	*Hermetia illucens*	Cuticle proteins	Unknown **	≈24	Karnaneedi et al. [109]
	*Hermetia illucens*	Odorant-binding proteins	Unknown **	≈14	Karnaneedi et al. [109]
	*Hermetia illucens*	Arginine kinase	Unknown **	Unknown	Broekman et al. [138]
	*Hermetia illucens*	Troponin	Unknown **	Unknown	Broekman et al. [138]
	*Hermetia illucens*	Triosephosphate Isomerase	Unknown **	Unknown	Broekman et al. [138]
	*Blattella germanica*	Bd 90 K	Bla g 1 *	45.8	WHO/IUIS (https://www.allergen.org) Allergome (https://www.allergome.org/index.php, accessed on 15 November 2025) Uniport (https://www.uniprot.org/)
	*Blattella germanica*	Inactive Aspartic Proteases	Bla g 2 *	38.5	WHO/IUIS (https://www.allergen.org) Allergome (https://www.allergome.org/index.php, accessed on 15 November 2025) Uniport (https://www.uniprot.org/)
	*Blattella germanica*	Hemocyanin	Bla g 3 *	78.7	WHO/IUIS (https://www.allergen.org) Allergome (https://www.allergome.org/index.php, accessed on 15 November 2025) Uniport (https://www.uniprot.org/)
	*Blattella germanica*	Calycin, lipocalin	Bla g 4 *	20.0	Chuang et al. [139]
	*Blattella germanica*	Glutathione S-transferase	Bla g 5 *	22.9	Chuang et al. [139]
	*Blattella germanica*	Troponin	Bla g 6 *	17.2	Hindley et al. [140]
	*Blattella germanica*	Tropomyosin	Bla g 7 *	32.8	Chuang et al. [139]
	*Blattella germanica*	Myosins	Bla g 8 *	22.	WHO/IUIS (https://www.allergen.org) Allergome (https://www.allergome.org/index.php, accessed on 15 November 2025) Uniport (https://www.uniprot.org/)
	*Blattella germanica*	Arginine Kinase	Bla g 9 *	39.7	WHO/IUIS (https://www.allergen.org) Allergome (https://www.allergome.org/index.php, accessed on 15 November 2025) Uniport (https://www.uniprot.org/)
	*Blattella germanica*	Serine protease	Bla g 10 *	26.3	WHO/IUIS (https://www.allergen.org) Allergome (https://www.allergome.org/index.php, accessed on 15 November 2025) Uniport (https://www.uniprot.org/)
	*Blattella germanica*	α-Amylase	Bla g 11 *	54.0	WHO/IUIS (https://www.allergen.org) Allergome (https://www.allergome.org/index.php, accessed on 15 November 2025) Uniport (https://www.uniprot.org/)
	*Blattella germ anica*	Chitinase	Bla g 12 *	58.0	Pomés et al. [141]
	*Blattella germanica*	Enolase	Bla g Enolase **	47.1	Chuang et al. [139]
	*Blattella germanica*	Vitellogenin	Bla g Vitellogenin **	213.5	Chuang et al. [139]
	*Blattella germanica*	Triosephosphate Isomerase	Bla g TPI **	26.8	Chuang et al. [139]
	*Blattella germanica*	Receptors for Activated Protein Kinase, RACK1	Bla g RACK1 **	35.7	Chuang et al. [139]
	*Periplaneta americana*	Cr-PII	Per a 1	44.5	WHO/IUIS (https://www.allergen.org) Allergome (https://www.allergome.org/index.php, accessed on 15 November 2025) Uniport (https://www.uniprot.org/)
	*Periplaneta americana*	Aspartic Protease	Per a 2 *	36.0	Gustchina et al. [142]
	*Periplaneta americana*	Hemocyanin	Per a 3 *	78.6	Wu et al. [143]
	*Periplaneta americana*	Lipocalin	Per a 4 *	17.0	Wangorsch et al. [144]
	*Periplaneta americana*	Glutathione-S-transferases	Per a 5 *	23.0	WHO/IUIS (https://www.allergen.org) Allergome (https://www.allergome.org/index.php, accessed on 15 November 2025) Uniport (https://www.uniprot.org/)
	*Periplaneta americana*	Troponin	Per a 6 *	17.1	Hindley et al. [140]
	*Periplaneta americana*	Tropomyosin	Per a 7 *	33.0	Asturias et al. [145]
	*Periplaneta americana*	Myosin	Per a 8 *	28.	Wangorsch et al. [144]
	*Periplaneta americana*	Arginine Kinase	Per a 9 *	43.0	Sookrung et al. [95]
	*Periplaneta americana*	Serine protease	Per a 10 *	28.0	Sudha et al. [146]
	*Periplaneta americana*	alpha-Amylase	Per a 11 *	55.	Fang et al. [147]
	*Periplaneta americana*	Chitinase	Per a 12 *	45.0	Fang et al. [147]
	*Periplaneta americana*	GAPDH	Per a 13 *	36.0	Xu et al. [148]
	*Periplaneta americana*	Glyceraldehyde-3-phosphate dehydrogenase	Per a 13 *	36.0	Xu et al. [148]
	*Periplaneta americana*	Enolase	Per a 14 *	50.0	Wang et al. [149]
	*Periplaneta americana*	Cytochrome C	Per a 15 *	15.0	Wang et al. [149]
	*Periplaneta americana*	Cofilin	Per a 16 *	20.0	Wang et al. [149]
	*Periplaneta americana*	Alpha-tubulin	Per a 17 *	53.0	Wang et al. [149]
	*Periplaneta americana*	Peptidyl-prolyl-cis-trans isomerase; Cyclophilin	Per a 18 *	24.0	Wang et al. [149]
	*Periplaneta americana*	Porin 3	Per a 19 *	7.4	Wang et al. [149]
	*Periplaneta americana*	Peroxiredoxin-6 (Prx6)	Per a 20 *	24.0	Wang et al. [149]
	*Periplaneta americana*	Fructose bisphosphate aldolase	Per a 21 *	39 kDa, 160 kDa	www.allergen.org
	*Periplaneta americana*	Pyruvate kinase	Per a 22 *	58 kDa, ~220 kDa	www.allergen.org
Mic roalgae	*Spirulina*	Superoxide dismutase (C3V3P3)	Unknown **	Unknown	Gromek et al. [34]
	*Spirulina*	GAPDH	Unknown **	Unknown	Gromek et al. [34]
	*Spirulina*	Triosephosphate Isomerase (D5A635)	Unknown **	Unknown	Gromek et al. [34]
	*Spirulina*	Thioredoxin reductase (D4ZSU6)	Unknown **	Unknown	Bianco et al. [120]
	*Spirulina*	Thioredoxin reductase (K1VP15)	Unknown **	Unknown	Bianco et al. [120]
	*Spirulina*	C-Phycocyanin-β-Subunit	Art pl beta_Phycocyanin **	18.0	Bianco et al. [120]
	*Chlorella*	Calmodulin proteins	Unknown **	Unknown	Bianco et al. [120]
	*Chlorella*	Troponin C	Unknown **	Unknown	Bianco et al. [120]
	*Chlorella*	Triosephosphate Isomerase	Unknown **	Unknown	Hamzelou et al. [150]
	*Chlorella*	Heat Shock Proteins	Unknown **	Unknown	Hamzelou et al. [150]
	*Chlorella*	Cyclic protein	Unknown **	Unknown	Hamzelou et al. [150]
	*Chlorella*	Fructose biphosphate aldolase	Unknown **	Unknown	Mariachiara et al. [120]
	*Bacteria bacillota*	Nattokinase (subtilisin-like serine protease)	Bac s 1 *	30.0	Suzuki et al. [151]
Bacte ria and fungi	*Saccharomyces cerevisiae*	Transaldolase	Sac c 14 **	37.0	Chou et al. [152]
	*Saccharomyces cerevisiae*	Carboxypeptidase	Sac c Carboxypeptidase Y **	59.8	WHO/IUIS (https://www.allergen.org)
	*Saccharomyces cerevisiae*	Cyclophilin	Sac c CyP **	≈18	Flückiger et al. [153]
	*Saccharomyces cerevisiae*	Enolase	Sac c Enolase **	46.8	WHO/IUIS (https://www.allergen.org) Allergome (https://www.allergome.org/index.php, accessed on 15 November 2025) Uniport (https://www.uniprot.org/)
	*Saccharomyces cerevisiae*	α-Glucosidase	Sac c Glucosidase **	68.1	WHO/IUIS (https://www.allergen.org) Allergome (https://www.allergome.org/index.php, accessed on 15 November 2025) Uniport (https://www.uniprot.org/)
	*Saccharomyces cerevisiae*	β-fructofuranosidase	Sac c Invertase **	60.0	WHO/IUIS (https://www.allergen.org) Allergome (https://www.allergome.org/index.php, accessed on 15 November 2025) Uniport (https://www.uniprot.org/)
	*Saccharomyces cerevisiae*	Manganese SO dismutase	Sac c MnSOD **	25.7	WHO/IUIS (https://www.allergen.org) Allergome (https://www.allergome.org/index.php, accessed on 15 November 2025) Uniport (https://www.uniprot.org/)
	*Saccharomyces cerevisiae*	Profiling	Sac c Profilin **	13.6	WHO/IUIS (https://www.allergen.org) Allergome (https://www.allergome.org/index.php, accessed on 15 November 2025) Uniport (https://www.uniprot.org/)
	*Saccharomyces cerevisiae*	Ribosomal Proteins	Sac c P2 **	10.7	WHO/IUIS (https://www.allergen.org) Allergome (https://www.allergome.org/index.php, accessed on 15 November 2025) Uniport (https://www.uniprot.org/)
	*Fusarium* spp.	Ribosomal Proteins P2	Fus c 1 *	11.0	Weber et al. [154]
	*Fusarium* spp.	Thioredoxin reductase	Fus c 2 *	13.0.	Weber et al. [154]
	*Fusarium* spp.	Transaldolase	Fus p 4 *	37.5	Weber et al. [154]
	*Fusarium* spp.	Vacuolar serine protease	Fus p 9 *	36.5	Yeh et al. [155]
	*Alternaria alternata*	Unknown	Alt a 1 *	16.4, 15.3	Abel-Fernández et al. [126]
	*Alternaria alternata*	Heat shock protein 70	Alt a 3 *	85.0	Abel-Fernández et al. [126]
	*Alternaria alternata*	Disulfide isomerase	Alt a 4 *	57.0	Abel-Fernández et al. [126]
	*Alternaria alternata*	Ribosomal protein P2	Alt a 5 *	11.0	Abel-Fernández et al. [126]
	*Alternaria alternata*	Enolase	Alt a 6 *	45.0	Abel-Fernández et al. [126]
	*Alternaria alternata*	Flavodoxin, YCP4 protein	Alt a 7 *	22.0	Abel-Fernández et al. [126]
	*Alternaria alternata*	Mannitol dehydrogenase	Alt a 8 *	29.0	Abel-Fernández et al. [126]
	*Alternaria alternata*	Aldehyde dehydrogenase	Alt a 10 *	53.0	Abel-Fernández et al. [126]
	*Alternaria alternata*	Aid ribosomal protein P1	Alt a 12 *	11.0	Abel-Fernández et al. [126]
	*Alternaria alternata*	Glutathione-transferase	Alt a 13 *	26.0	Abel-Fernández et al. [126]
	*Alternaria alternata*	Manganese SO dismutase	Alt a 14 *	24.0	Abel-Fernández et al. [126]
	*Alternaria alternata*	Vacuolar serine protease	Alt a 15 *	58.0	Abel-Fernández et al. [126]

## 3. Influence of Food Processing Techniques on Protein Sensitization

**Figure 4:**
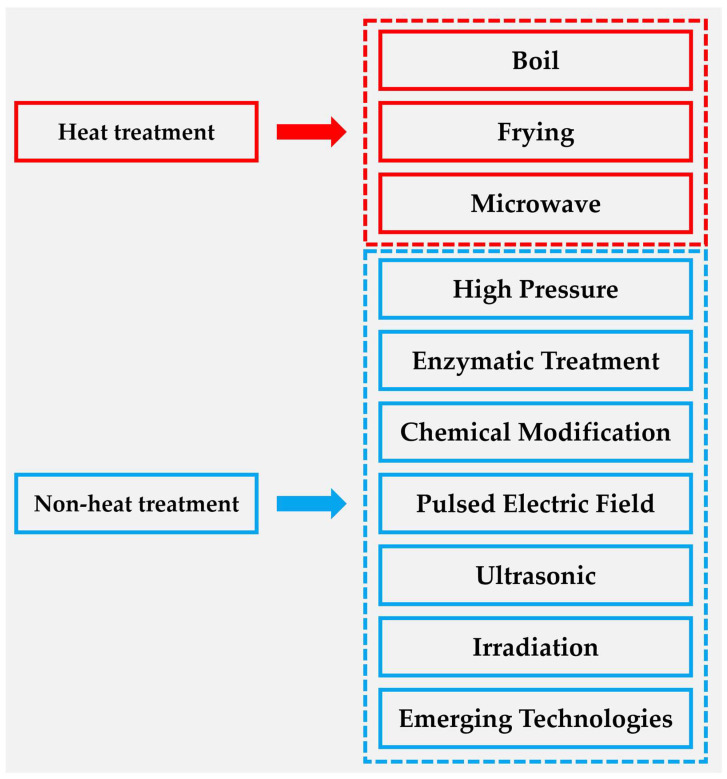
Food processing methods can be classified according to the heat effect: heat treatment methods include boiling, frying and microwave; non-heat treatment methods include high pressure, enzyme treatment, chemical modification, pulsed electric field, ultrasound, irradiation and emerging methods.

Food allergies typically arise from abnormal immune system responses to specific proteins, triggering a spectrum of clinical symptoms ranging from mild discomfort to severe anaphylactic reactions that can be life-threatening. The mechanisms underlying protein allergenicity are complex and involve antigen recognition, immune activation, and the subsequent inflammatory responses mediated by these processes. Recent studies have demonstrated that appropriate food processing techniques-including thermal treatment, enzymatic modification, fermentation, high-pressure processing, and plant polyphenol conjugation-can significantly reduce the cross-reactivity and allergenic potential of allergenic proteins (Figure 4) [131,137,138,156,157,158]. These processing methods can alter the structural conformation of allergenic proteins, thereby reducing the availability of IgE-binding epitopes. Since the allergenicity of a protein is largely determined by its capacity to bind IgE antibodies, diminishing the number of accessible IgE-binding sites through structural modification can effectively decrease the protein’s immunoglobulin E affinity, ultimately mitigating its allergenic potential.

### 3.1. Heat Treatment

#### 3.1.1. Boiling and Frying

Thermal processing represents a commonly employed method for reducing the allergenicity of allergenic proteins, as high-temperature treatments induce conformational changes in protein spatial structures that may either mask or destroy IgE-binding epitopes (thereby reducing allergenicity) or conversely expose new epitopes (thereby increasing allergenicity) (Table 3) [159,160,161]. He et al. investigated the effects of heating on the allergenicity of silkworm pupa protein extracts [162], demonstrating that protein bands corresponding to allergens in the 43–90 kDa range disappeared after heating above 80 °C, while bands at 25–33 kDa remained clearly visible, indicating that heating reduces but does not completely eliminate the allergenicity of silkworm pupa protein extracts. This phenomenon was further corroborated by Lamberti et al. [108]. He et al. also observed that heating induced alterations in the higher-order structures of silkworm pupa protein extracts, such as reduced α-helix and β-sheet contents compared to non-heated extracts, which exposed hydrophobic regions that could either mask or expose epitopes, thereby affecting IgE-binding capacity. Moreover, protein degradation and aggregation occurring at heating temperatures above 80 °C further diminished allergenicity. Broekman et al. examined the effects of boiling (100 °C for 1 or 10 min), electric stove roasting (1000 W for 3.5 min), and frying (180 °C for 30 s) on mealworm allergenicity [138], finding that none of these treatments significantly altered IgE-binding or cross-linking capacities, but merely changed the solubility of mealworm proteins without affecting allergenicity. This solubility change likely resulted from heat-induced structural modifications; tropomyosin (the primary mealworm allergen), as a muscle protein composed of highly α-helical monomers assembled into rod-shaped coiled-coil dimers, possesses a complex structure conferring strong thermal stability that resists heat-induced structural disruption and consequent changes in allergenicity, explaining why thermal processing failed to significantly affect mealworm allergen allergenicity [163]. Subsequently, Lamberti et al. treated mealworms with boiling (100 °C for 5 min) and frying (180 °C for 3 min), comparing their allergenicity to untreated controls [108], and found that high-temperature frying partially reduced cross-reactivity of mealworm protein extracts without completely eliminating allergenicity. Compared to Broekman et al.’s shorter frying time (180 °C for 30 s), Lamberti et al.’s longer frying duration (180 °C for 3 min) appeared more effective at reducing mealworm allergen allergenicity, potentially explaining why frying is more commonly applied to mealworm processing. Fried locusts represent another common insect food product; Phiriyangkul et al. found that frying (180 °C for 3 min) significantly reduced allergenicity of arginine kinase and enolase in locusts while paradoxically increasing that of glyceraldehyde-3-phosphate dehydrogenase [136]. Interestingly, different thermal processing methods applied to the same allergenic protein may yield diametrically opposed results. Lamberti et al. treated locusts with both boiling (100 °C for 5 min) and frying (180 °C for 3 min) [108], finding that fried locust proteins exhibited cross-reactivity reduced to 29% of the original level, whereas boiled locust proteins not only failed to reduce but actually increased cross-reactivity. This discrepancy likely arises because frying induces irreversible denaturation of locust tropomyosin, eliminating IgE-binding activity, while boiling causes partial denaturation that exposes previously hidden epitopes, enhancing IgE-binding activity. Zhang et al. subjected peanut allergen Ara h 2 to both boiling and roasting treatments, observing that boiling reduced allergenicity while roasting enhanced allergic reactions [164]. Similarly, Li et al. found that 1 h heating of β-conglycinin (Gly m 5) increased its allergenicity [165]. In summary, while heating generally reduces allergenicity of allergenic proteins, its effectiveness is significantly influenced by protein structure, with instances where allergenicity remains unchanged or even increases post-heating. For edible insects, heating or boiling may be more suitable for processing less stable allergens, whereas more extreme treatments like high-temperature frying effectively disrupt higher-order protein structures, proving particularly effective against thermostable allergens such as myosin and troponin.

**Table 3:** Summary of the Effects of Heat Treatment on the Allergenicity of Selected Proteins.

Allergen Source	Heat Treatment	Effect on Allergenicity	Key Determining Factor
Silkworm pupa	Heating > 80 °C	Decreased	Protein degradation/aggregation; structural unfolding leading to epitope masking.
Silkworm pupa	Heating > 80 °C	Unchanged/Partial decrease	Thermostability of specific protein components.
*Tenebr io molitor*	Boiling (100 °C, 1–10 min), Frying (180 °C, 30 s)	Largely unchanged	High intrinsic thermal stability of coiled-coil structure.
*Tenebrio molitor*	Frying (180 °C, 3 min)	Decreased (Partial)	Longer duration/intensity of heating leading to irreversible denaturation.
*Locust*	Frying (180 °C, 3 min)	Decreased	Heat-labile structure; denaturation destroys epitopes.
*Locust*	Frying (180 °C, 3 min)	Increased	Exposure of previously hidden (cryptic) epitopes due to unfolding.
*Locust*	Frying (180 °C, 3 min)	Decreased	Irreversible denaturation eliminating IgE-binding
*Locust*	Boiling (100 °C, 5 min)	Increased	Partial denaturation exposing hidden epitopes.
Peanut	Boiling	Decreased	Leaching of allergens into water; structural changes.
Peanut	Roasting	Increased	Maillard reaction creating new or stabilizing existing epitopes.
Soybean	Heating (1 h)	Increased	Aggregation or stabilization of conformational epitopes.

#### 3.1.2. Microwave

Microwave treatment may significantly affect the activity and structural properties of proteins and peptides, including unfolding of tertiary structures and alterations in secondary structures [166,167].Microwave is a commonly employed food processing method, as proteins and peptides possess higher dielectric constants, making them particularly susceptible to structural and functional alterations induced by microwaves [167]. Dong et al. investigated the effects of microwave treatment on the allergenicity of shrimp allergenic proteins [168], finding that the lowest intensity of tropomyosin bands was observed after treatment at 125 °C for 10 min.

### 3.2. Non-Heat Treatment

#### 3.2.1. High Pressure

High-pressure processing is widely applied in food manufacturing. This treatment may alter the spatial conformation of allergenic proteins, disrupting the integrity of their higher-order structures. This disruption can lead to the complete exposure or masking of antibody-binding epitopes, thereby affecting the allergenicity of these proteins [169,170]. Studies have demonstrated that high-pressure treatment can effectively reduce the allergenicity of edible insect allergenic proteins. Li et al. found that 200 MPa pressure caused deformation of myosin heavy chain while 500 MPa pressure led to significant degradation of troponin [171]. Chen et al. investigated the effects of high-temperature-high-pressure treatment on the allergenicity of tropomyosin in ready-to-eat clam meat [172], showing that extended processing time improved both flavor and texture of the clam meat while significantly reducing the allergenicity of clam tropomyosin. Martínez-Maldonado et al. observed that myosin heavy chain underwent unfolding and aggregation under 600 MPa pressure, which likely resulted in epitope destruction and subsequent changes in allergenicity.

#### 3.2.2. Enzymatic Treatment

Enzymatic treatment represents another common approach for reducing allergen immunogenicity. Enzyme treatment to reduce the allergenicity of allergenic proteins generally involves two processes. The first process is enzymatic hydrolysis, wherein proteases hydrolyze proteins and disrupt their spatial structure and linear epitopes, thereby achieving a reduction in allergenicity [173]. Another method is enzyme cross-linking, which involves intramolecular or intermolecular cross-linking reactions induced by enzymes that alter protein molecular weight. This induces protein aggregation and disrupts secondary structures and IgG/IgE binding epitopes [174]. He et al. investigated the effects of pepsin on the allergenicity of silkworm pupa proteins [162], demonstrating that pepsin treatment reduced the allergenicity of silkworm pupa proteins to a certain extent, although 25–33 kDa allergenic proteins still exhibited resistance to pepsin digestion, indicating that these allergens in silkworm pupa protein extracts possess both thermal stability and partial resistance to enzymatic degradation. Notably, low-molecular-weight fragments generated from silkworm proteolysis may retain allergenic potential, particularly proteins in the 25–33 kDa range. Enzymatic hydrolysis also shows some efficacy in attenuating allergenicity of mealworm and cricket allergens. Hall et al. examined the impact of alkaline protease treatment on cricket allergenicity [175], finding that enzymatic hydrolysis significantly affected the allergenicity of cricket proteins, especially tropomyosin. Untreated cricket proteins and protein hydrolysates (CPH) with 15–50% degree of hydrolysis exhibited strong reactivity with anti-tropomyosin antibodies in immunoblotting, whereas CPH with 60–85% hydrolysis showed negligible reactivity, suggesting that higher degrees of hydrolysis significantly reduce protein allergenicity, likely through conformational changes in tropomyosin that diminish or eliminate its antigenicity. Shen et al. conducted similar studies [176], showing that α-chymotrypsin treatment markedly reduced tropomyosin allergenicity, with nearly complete loss of immunoreactivity after 240 min of continuous treatment. Although enzymatic hydrolysis is highly effective in reducing allergenicity, its specificity prevents targeted modification of IgE-binding epitopes, and enzymatic action may even expose additional epitopes [177,178], necessitating the combination of enzymatic hydrolysis with other processing methods. Hall and Liceaga demonstrated that microwave-assisted enzymatic hydrolysis significantly reduced IgE-binding capacity of cricket allergens [179]. Boukil et al. employed high hydrostatic pressure-assisted enzymatic hydrolysis for mealworm powder processing [180], finding that high hydrostatic pressure disrupted protein tertiary structures, facilitated enzyme access to proteins, and enabled more efficient hydrolysis into peptides, thereby reducing allergenicity. However, Clemente et al. observed increased antigenicity in chickpea proteins after flavor protease hydrolysis [181], and Cabanillas et al. noted increased IgE reactivity detected by ELISA at 30 min when using pepsin alone to treat roasted peanut proteins [182]. In another study, Sung et al. found that while enzymatic hydrolysis with bromelain slightly reduced buckwheat protein allergenicity, subsequent treatment with pepsin and GC106 actually increased allergenicity [183]. Moreover, as insect proteins inherently possess earthy flavor profiles, Grossmann et al. examined flavor changes in mealworm proteins before and after enzymatic hydrolysis [184], revealing that enzymatic hydrolysis significantly enhanced both bitterness and umami flavors [161]. Therefore, when applying enzymatic hydrolysis to allergenic proteins, comprehensive consideration of factors including allergenicity and sensory evaluation is essential.

#### 3.2.3. Chemical Modification

Glycosylation of proteins in food refers to the non-enzymatic chemical reaction between amino compounds in proteins (primarily lysine with ε-amino groups) and carbonyl compounds (mainly reducing sugars) [174]. Protein glycosylation, which involves the reaction of proteins with carbohydrate compounds to attach sugar chains to amino acid residues, can lead to the destruction or masking of allergenic epitopes. Zhang et al. investigated the glycosylation reactions of tropomyosin with carbohydrates of varying molecular weights [185], finding that glycosylation with glucose, maltotriose, maltopentaose, and maltulose significantly disrupted allergenic epitopes and reduced allergenicity, whereas glycosylation with maltose showed no significant effect on reducing tropomyosin allergenicity. Han et al. examined the impact of the Maillard reaction on the allergenicity of tropomyosin and arginine kinase [186], demonstrating that the Maillard reaction significantly reduced the allergenicity of both TM and AK, particularly when arabinose was used as the reducing sugar. The Maillard reaction caused a reduction in α-helices and an increase in β-turns and random coils in the structures of tropomyosin and arginine kinase, leading to the masking or destruction of epitopes and consequent reductions in allergenicity.

#### 3.2.4. Pulsed Electric Field

Pulsed electric field (PEF) represents a novel non-thermal food sterilization technology that processes liquid and semi-solid foods using high electric field strengths (10–50 kV/cm), short pulse widths (0–100 μs), and high pulse frequencies (0–2000 Hz). Researchers have begun investigating the effects of PEF technology on the allergenicity of algal-derived nutrients and bioactive substances. Polikovsky et al. compared proteins extracted from *Ulva* treated with PEF versus those processed using thermochemical methods or without PEF treatment [187]. The study found that PEF selectively prevented the release of calmodulin, a major allergenic protein in algae, whereas non-PEF-treated samples contained identifiable Superoxide dismutase (SOD), and thermochemically treated samples revealed four potential allergens including SOD, Troponin C, Aldolase A, and Thioredoxin reductase. These results suggest that PEF can reduce the release of certain known allergens during extraction, potentially offering a safer method for extracting edible algal proteins.

#### 3.2.5. Ultrasonic

High-intensity ultrasound, an efficient food processing and preservation technique, has been successfully applied in homogenizing emulsions, enzyme inactivation, assisted extraction processes, accelerating dehydration, and expediting aging and maturation procedures [188,189]. Ultrasonic treatment generates intense shear forces and temperature fluctuations through acoustic vibration and cavitation, thereby altering the spatial structure of allergenic proteins. This process disrupts or conceals the antibody-binding epitopes of allergenic proteins, consequently reducing their allergenicity [174]. Li et al. utilized high-intensity ultrasound to treat shrimp allergenic proteins [190], demonstrating that high-intensity ultrasonic treatment significantly reduced the allergenicity of shrimp tropomyosin, with the treated tropomyosin exhibiting markedly diminished IgE-binding capacity compared to untreated controls. Ultrasound treatment induced structural changes in the proteins, including the unfolding of secondary structures that may lead to either exposure or obliteration of allergenic epitopes, thereby influencing immune system recognition and response to these proteins. Furthermore, the study revealed that combining heat treatment with ultrasound produced enhanced effects, potentially attributable to the thermal effects initiated by ultrasonic waves. Under high-temperature conditions, the reversible unfolding of proteins and rearrangement of disulfide bonds may alter or partially inactivate allergenic epitopes, consequently reducing allergenicity.

#### 3.2.6. Irradiation

Irradiation technology is not only an environmentally friendly, low-carbon, and highly efficient method for eliminating allergenicity, but also effectively preserves the flavor and quality of food [191]. Irradiation generates free radicals through radiolysis, which can disrupt the spatial conformation and epitope structure of allergenic proteins, leading to their denaturation, aggregation, or fragmentation [192]. This conformational change may mask or disrupt antigenic epitopes in allergenic proteins that are recognized by the immune system, thereby reducing their allergenicity. Ji-Hyun Seo et al. irradiated Ovalbumin with gamma rays at doses of 50 kGy and 100 kGy, respectively. They found that compared to groups attacking non-irradiated ovalbumin, mice treated with irradiated ovalbumin exhibited significantly reduced total IgE levels in serum [192]. This indicates irradiation effectively diminishes ovalbumin’s sensitizing capacity. Meng et al. measured antibody binding capacity and histamine release capacity after gamma irradiation of α-lactalbumin [193]. The study showed that after 10 kGy irradiation, the antibody binding capacity of α-lactalbumin decreased by approximately 95%, histamine release capacity decreased by approximately 26%, and sensitization potential was significantly reduced. Zhu et al. measured antibody binding capacity and immunoblotting signals in porcine serum albumin after gamma irradiation at 1 kGy and 8 kGy, respectively [194]. Results indicated that antibody binding capacity of irradiated pig serum albumin decreased by over 90% compared to the untreated group, while Western blot analysis showed progressively lighter signal band coloration with increasing irradiation intensity. This demonstrates a significant reduction in protein allergenicity. Additionally, Liu et al. observed that high-dose electron beam irradiation (10 kGy) of frozen shrimp allergenic proteins significantly reduced protein allergenicity [195]. However, allergenicity increased after low-dose irradiation, likely due to structural changes that increased antibody-binding epitopes. Low-dose irradiation may enhance allergenicity, while higher doses effectively reduce allergens but may adversely affect food flavor and quality. Therefore, controlling irradiation dose is a critical step in using irradiation for desensitization processes [174].

#### 3.2.7. Emerging Technologies

Beyond traditional processing methods such as boiling, frying, and high-pressure treatment, numerous emerging technologies have been developed to reduce the allergenicity of allergenic proteins. Cold atmospheric pressure plasma (CAPP) technology represents a non-thermal method for food preservation [196]. CAPP can reduce allergenicity by disrupting the structural conformation of allergenic proteins, particularly through the reduction of IgE-binding epitopes. Research has demonstrated CAPP’s effectiveness in attenuating allergenicity; for instance, direct dielectric barrier discharge plasma treatment reduced the allergenicity of shrimp tropomyosin by 60% [160,197]. Furthermore, studies have shown that algal allergens exhibit significant resistance to conventional heat treatment and enzymatic hydrolysis methods for reducing allergenicity [198], suggesting that CAPP may hold particular promise for mitigating algal allergenicity. However, research on the application of CAPP specifically targeting protein allergenicity remains limited and requires further in-depth investigation. Beyond its potential for allergen reduction, CAPP has also been utilized to produce high-quality insect powders [199]. If this technology were to be more comprehensively studied, it is conceivable that it could simultaneously enhance the quality of insect powders while reducing the allergenicity of insect allergenic proteins, which would have significant positive implications for sustainable development and food safety.

Li et al. developed a magnetic nanocomposite with photochemical synergistic capabilities to reduce the allergenicity of phospholipase A2 (PLA2) [200]. The photochemical dynamic interactions induced structural alterations in PLA2, specifically transforming its α-helical conformation into β-sheet structures, thereby diminishing its IgE-binding capacity and mitigating the risk of allergic reactions. Furthermore, owing to the magnetic properties of the nanocomposite, the treated PLA2 could be effectively adsorbed and separated using an external magnetic field, ensuring the safety of the processed product.

The contents of Table 4 are summarized from the preceding sections.

### 3.3. Challenges and Future Directions of Processing Technology Applications

Current research predominantly focuses on how processing methods influence the allergenicity of edible insect allergens, whereas studies investigating the impact of processing on the allergenicity of algal and microbial (bacterial and fungal) allergens remain limited. Yeast proteins are generally considered hypoallergenic [201]; however, there have been documented cases of yeast proteins inducing allergic reactions. Regarding *S. cerevisiae*, thermal processing appears to be a potentially effective method for reducing allergenicity. A clinical case report described a patient who experienced severe allergic reactions to *S. cerevisiae* extracts yet tolerated *S. cerevisiae* present in baked goods such as bread [202]. This phenomenon was hypothesized to result from thermal denaturation of allergenic proteins during baking, which reduced the allergenic potential. Additionally, research on processing modifications of algal allergenic proteins remains limited, primarily due to the pivotal role of phycocyanin as the principal allergen in algae; processing methods that compromise the structural integrity and functional properties of phycocyanin would significantly diminish the nutritional value of algal proteins. Therefore, against the backdrop of security challenges in sustainable protein resource development, systematically evaluating the impact of processing on sensitization to algal and microbial allergens represents a critical scientific issue that demands urgent priority.

To address these challenges, future research should focus on several key areas. First, comprehensive identification and characterization of allergens in microalgae and microorganisms should be conducted using proteomics and bioinformatics technologies. Standardized IgE panels should be established using serum samples from sensitized individuals to lay the foundation for allergy risk assessment. Building on this, for algal allergens, a comprehensive investigation of the structure-function relationships of key sensitizing components like phycocyanin is needed. The focus should be on exploring non-thermal processing technologies (such as cold plasma or pulsed electric fields) or targeted enzymatic hydrolysis techniques to achieve precise destruction of IgE-binding epitopes while preserving nutritional value. For microbial protein sources like yeast, strategies may include: developing hypoallergenic strains through genetic engineering or transgenic techniques, or employing targeted protein extraction during processing to selectively degrade allergenic proteins and reduce sensitization risks. Finally, any effective desensitization processing technology must undergo validation through clinical or animal trials. Therefore, individuals with a history of allergies to algae or fungi should continue to avoid consuming related products as a precautionary measure to safeguard health until more comprehensive safety solutions are established.

Food processing technologies exhibit diverse potential in modulating the allergenicity of allergenic proteins, yet their efficacy is highly contingent upon the intrinsic properties of the allergens (such as structural stability and epitope distribution) and the optimization of processing parameters. Thermal processing, while capable of disrupting certain IgE-binding epitopes by altering protein conformation, demonstrates limited effectiveness against thermally stable allergens (e.g., tropomyosin) and may even enhance allergenicity through epitope exposure under certain conditions. Enzymatic hydrolysis requires precise control of hydrolysis degree or integration with complementary treatments (such as high pressure or microwave) to achieve effective allergen degradation; however, the inherent specificity of enzymes and the potential risk of epitope exposure necessitate careful evaluation. Physical methods including high pressure, microwave, and ultrasound disrupt protein tertiary structures to reduce allergenicity, while emerging technologies such as pulsed electric fields and cold atmospheric plasma offer novel approaches by selectively inhibiting allergen release through non-thermal mechanisms, thereby providing innovative pathways for developing hypoallergenic foods. Notably, different processing methods may exert opposite effects on the same allergen (e.g., the contrasting impacts of boiling versus frying on grasshopper allergenicity), underscoring the necessity of tailoring processing strategies based on allergen-specific characteristics. Future research should prioritize elucidating the structure-processing response relationships of allergens, developing integrated multi-technology solutions, and extending these strategies to emerging protein sources such as algae and microorganisms, while simultaneously balancing the comprehensive impacts on nutritional quality, sensory attributes, and safety. Such advancements will provide critical scientific support for the development of sustainable, low-allergenic protein resources.

## 4. Conclusions and Prospects

As global population growth and resource pressures intensify, the development of sustainable alternative protein sources has become a key strategy to address protein supply issues. This paper systematically reviews the nutritional value, potential allergens and their sensitising mechanisms of alternative proteins such as edible insects, microalgae and microorganisms (fungi, bacteria, yeasts) and explores the role of different processing technologies in reducing the risk of allergens.

Edible insects, microalgae, and microorganism proteins, with their high nutritional density and low environmental dependence, are gradually emerging as a crucial supplement to traditional animal proteins. Edible insects such as *T. molitor* and *B. mori* contain protein levels of 50–70%, along with abundant essential amino acids and minerals; microalgae like *A. platensis* produce phycocyanin with both nutritional and functional properties; and microorganisms including *S. cerevisiae* and *F. venenatum* manufacture proteins with high bioavailability through efficient fermentation. This diversity of protein sources provides the food industry with abundant options, yet their widespread application remains restricted by allergenic risks. Allergenic proteins commonly present in edible insects, microalgae, and microorganism proteins include Tropomyosin and Arginine Kinase in insects, phycocyanin in microalgae, and Ribosomal Protein P2 in fungi. These allergens can not only trigger local or systemic allergic reactions (such as asthma and anaphylactic shock) but also exhibit significant cross-reactivity with traditional allergens like crustaceans and mites. For example, the Tropomyosin in *H. illucens* shares 75–80% sequence homology with shrimp allergens, posing high risks for seafood-allergic individuals.

The application of processing technologies such as thermal treatment, enzymatic hydrolysis, high-pressure processing, and glycosylation can partially reduce the IgE-binding capacity of food allergens. Various processing techniques demonstrate significant potential in reducing the allergenicity of microbial and edible insect proteins. For example, high-temperature deep-frying at 180 °C significantly reduces the allergenicity of *T. molitor* and *L. migratoria* allergens by destroying the advanced protein structure, and enzymatic hydrolysis (such as treatment with alkaline protease) can degrade the epitopes of Tropo-myosin in *A. domesticus*, while non-thermal technologies (such as cold atmospheric plasma) show potential by selectively inhibiting the release of allergens. However, the effectiveness of these methods is highly dependent on the inherent properties of specific allergens. The key challenge in heat treatment lies in the thermal stability of proteins such as actin, which may reduce the effectiveness of heat processing. In certain cases, it can even expose hidden epitopes, thereby enhancing allergenicity. Additionally, various desensitization processing techniques may be accompanied by nutrient loss or deterioration of sensory characteristics. For instance, processes like enzymatic hydrolysis may generate bitter peptides or compromise sensory properties.

In summary, future research should prioritize expanding allergen protein databases by comprehensively identifying sensitizing proteins through proteomics and bioinformatics tools. Secondly, novel desensitization techniques or optimized composite treatment methods should be developed to achieve efficient desensitization while preserving the nutritional and sensory qualities of food. Additionally, targeted processing techniques for allergen reduction should be developed, aiming to minimize the impact on sensory characteristics or nutritional value while reducing the allergenicity of allergenic proteins.

## Figures and Tables

**Table 1:** Applications of Insect Protein and Microbial Protein in Food Products.

Food	Added	Advantages	Disadvantages	Ref
Emulsified Sausage	*T. molitor* *B. mori*	Increased protein content; Improved cooking yield; Enhanced hardness (chewiness); Reduced moisture content;	Increased fat content; Darker color; Reduced antioxidant activity	[55]
Frankfurters	*T. molitor* L.	Increased protein content; Increased mineral content; Reduced fat content	Reduced moisture content; Decreased meat batter solubility; Reduced hardness (chewiness); Decreased emulsion stability; Lower acceptability	[56]
Meat Batter	*B. mori*	Increased protein content; Increased mineral content; Enhanced hardness (chewiness); Reduced cooking loss	Lower sensory acceptability	[57]
Tteokgalbi	*A. dichotoma* *P. brevitarsis*	Improved antioxidant activity Increased cooking yield Softer texture	Softer texture leading to poorer mouthfeel	[58]
Sausages	*P. sapidus*	Improved nutritional value; Higher sensory acceptability; Lower production cost; Environmentally friendly	Reduced hardness and crispiness; Less vibrant color; Flavor requires further improvement	[59]
Cooked ham	*S. cerevisiae*	Increased protein content’ Increased mineral content; Enhanced nutritional properties; Improved texture	Extended cooking time may increase production costs	[60]
Beef burger	*S. cerevisiae*	Increased protein content; Improved cooking yield; Lower production cost	Deteriorated sensory characteristics Lower acceptability	[61]
Beef meatball	Yeast	Improved nutritional value; Enhanced flavor and texture; Increased cooking yield	Affected beef meatball color	[62]
Cookie	*T. monitor* *Z. atratus*	Increased protein content; Increased mineral content; Reduced carbohydrate content; Darker cookie color; Increased moisture content	Increased cookie hardness; Reduced sensory attributes; Lower acceptability	[63]
Cookie	*T. molitor*	Increased protein content; Increased fat content; Higher polyunsaturated fatty acid content	Lower acceptability	[64]
Cookie	*R. differens*	Improved protein digestibility; Richer volatile compounds	Harder texture; Lower acceptability	[65]
Cookie	*A. domesticus*	Increased protein content; Increased moisture content	Lower acceptability	[66]
Cookie	*G. belina*	Increased protein content; Increased fat content; Increased mineral content	Lower acceptability	[67]
Cookie	*S. cerevisiae*	Increased protein content; Increased fiber content; Improved antioxidant capacity; Reduced processing cost	Reduced sensory attributes; Lower acceptability	[68]
Muffins	*L. migratoria* *T. molitor*	Increased protein content; Increased fat content; Reduced hardness	Lower sensory attributes	[69]
Nut Bar	*A. domesticus* L. *A. diaperinus* P. *T. molitor* L.	Improved antioxidant activity; Higher phenolic compound content; Increased phytosterol content	Lower sensory attributes; Lower consumer acceptability	[70]
Cereal bar	*A. domesticus* *G. sigillatus*	Increased protein content; Increased mineral content; Improved sensory; characteristics	Lower market acceptability; Affected flavor, aroma, appearance, and texture	[71]
Erişte	*T. molitor* *L. migratoria*	Increased protein content; Increased mineral content; Increased crude fiber content	Reduced noodle expansion volume; Reduced brightness and yellowness; Increased cooking loss; Reduced moisture absorption; Deteriorated texture and sensory characteristics	[72]
Pasta	*A. domesticus*	Increased protein content; Increased fat content; Increased mineral content; Increased energy value; Reduced cooking loss; Improved hardness (chewiness)	Reduced noodle brightness; Undesirable off-flavors developed	[73]
Pasta	*B. mori*	Increased protein content	Darker noodle color; More brittle texture; Increased cooking loss; Compromised structural integrity	[74]
Pasta	*H. illucens* *B. mori*	Increased protein content; Increased mineral content; Improved hardness (chewiness)	Reduced noodle brightness; Increased cooking loss	[75]
Bread	*L. migratoria*	Increased protein content; Increased fat content; Increased crude fiber content; Improved functional properties (water absorption, oil absorption, foaming, emulsification)	Reduced bread volume; Darker bread color; Lower acceptability	[76]

**Table 4:** Advantages and limitations of processing technology.

Processing Technology	Advantages	Limitations
Boiling/Frying	Reduces allergenicity of most proteins; Frying is particularly effective for thermostable proteins	May expose new IgE-binding epitopes; Ineffective for certain proteins; High temperatures may degrade nutritional quality
High-Pressure	Destroys IgE-binding sites; Improves food texture	Requires high-pressure equipment; Potential alterations in sensory attributes (e.g., taste, texture)
Microwave	Fast and efficient; Exhibits synergistic effects when combined with other technologies	Risk of protein over-denaturation; High operational costs for industrial applications
Ultrasound	Significantly disrupts protein structures; Compatible with other processing methods	Potential generation of free radicals; Expensive large-scale implementation
Enzymatic Hydrolysis	Degrades allergens; Enhances protein solubility	Short peptides may retain allergenicity; Altered food flavor profiles
Chemical Modification	Masks allergenic epitopes; Improves protein stability	Ineffective for specific conditions; Possible impacts on nutritional value
Pulsed Electric Field	Non-thermal treatment; Selectively inhibits allergen release	High equipment costs; limited research on broad applicability
Irradiation	Environmentally friendly; Efficient and low-carbon	Low-dose irradiation may enhance sensitization; High-doses may affect the flavor and quality of food
Cold Atmospheric Pressure Plasma	Thoroughly destroys IgE-binding epitopes; Preserves nutritional value	Laboratory-scale validation only; Mechanisms require further elucidation
Magnetic Nanocomposite Adsorption	Efficiently separates allergenic proteins	Complex technology; Challenges in scaling up for industrial production

## Data Availability

No new data were created or analyzed in this study. Data sharing is not applicable to this article.
